# Technology-Assisted Mental Health Intervention Delivered by Frontline Workers at Community Doorsteps for Reducing Anxiety and Depression in Rural Pakistan: Protocol for the mPareshan Mixed Methods Implementation Trial

**DOI:** 10.2196/54272

**Published:** 2024-07-23

**Authors:** Fauziah Rabbani, Javeria Nafis, Samina Akhtar, Muhammad Shahid Khan, Saleem Sayani, Amna Siddiqui, Sameen Siddiqi, Zul Merali

**Affiliations:** 1 Brain and Mind Institute Aga Khan University Karachi Pakistan; 2 Department of Community Health Sciences Aga Khan University Karachi Pakistan; 3 Digital Health Resource Centre Aga Khan Development Network Karachi Pakistan

**Keywords:** anxiety, depression, feasibility, mental health, health workers, mPareshan app, implementation research

## Abstract

**Background:**

There is a dearth of specialized mental health workforce in low- and middle-income countries. Use of mobile technology by frontline community health workers (CHWs) is gaining momentum in Pakistan and needs to be explored as an alternate strategy to improve mental well-being.

**Objective:**

The aim of this study is to assess the feasibility, acceptability, and usefulness of an app-based counseling intervention delivered by government lady health workers (LHWs) to reduce anxiety and depression in rural Pakistan.

**Methods:**

Project mPareshan is a single-arm, pre- and posttest implementation research trial in Badin District, Sindh, using mixed methods of data collection executed in 3 phases (preintervention, intervention, and postintervention). In the preintervention phase, formative qualitative assessments through focus group discussions and in-depth interviews assess the acceptability and appropriateness of intervention through perceptions of all concerned stakeholders using a specific interview guide. A REDCap (Research Electronic Data Capture)-based baseline survey using Patient Health Questionnaire-9 (PHQ-9) and Generalized Anxiety Disorder-7 Scale (GAD-7) determines the point prevalence of depression and anxiety among consenting men and women older than 18 years. Individuals with mild and moderate anxiety and depression are identified as screen positives (SPs) and are eligible for mPareshan app–based intervention. Mental health literacy of health workers is improved through customized training adapting the World Health Organization’s Mental Health Gap Action Programme guide 2.0. The intervention (mPareshan app) consists of tracking, counseling, and referral segments. The tracking segment facilitates participant consent and enrollment while the referral segment is used by LHWs to transfer severe cases to the next level of specialist care. Through the counseling segment, identified SPs are engaged during LHWs’ routine home visits in 6 face-to-face 20-minute counseling sessions over 6 months. Each session imparts psychoeducation through audiovisual aids, breathing exercises, and coping skills to reduce stress. Clinical and implementation outcomes include change in mean anxiety and depression scores and identification of facilitators and barriers in intervention uptake and rollout.

**Results:**

At the time of this submission (April 2024), we are analyzing the results of 366 individuals who participated in the baseline prevalence survey, the change in knowledge and skills of 72 health workers who took the mPareshan training, change in anxiety and depression scores of 98 SPs recruited for app-based counseling intervention, and perceptions of stakeholders pre- and postintervention gathered through 8 focus group discussions and 18 in-depth interviews.

**Conclusions:**

This trial will assess the feasibility of early home-based mental health screening, counseling, and prompt referrals by frontline health workers to reduce anxiety and depression in the community. The study findings will set the stage for integrating mental health into primary health care.

**Trial Registration:**

Australian New Zealand Clinical Trial Registry ACTRN12622000989741; https://tinyurl.com/5n844c8z

**International Registered Report Identifier (IRRID):**

DERR1-10.2196/54272

## Introduction

### Background

Mental disorders account for 14% of the global burden of disease [1]. Globally, 322 million (4.4%) and 264 million (3.6%) people endure depression and anxiety, respectively [2]. The data from World Mental Health Surveys concluded that people with mental disorders sought treatment in a very small proportion, particularly in low- and-middle income countries (LMICs) [3]. Barriers to accessing mental health care facilities in LMICs include the cost of mental health care, poor distribution of available resources, and the distance to reach a mental health facility [4,5].

Pakistan has a sizable burden of psychiatric morbidity, particularly depression and anxiety. It is an LMIC with a population of 220 million, with almost 60% residing in rural areas [6,7]. Pakistan has the highest depression rates in contrast to other transitional countries which can be attributed to several factors such as poverty, political turmoil, gender inequality, and natural disasters, to mention a few [1,5,7,8]. The COVID-19 pandemic has worsened the mental health crisis with a rise in depression, anxiety, and stress [9]. The number of deaths by suicide has also increased since the pandemic [10].

Community health workers (CHWs) are the first contact for individuals seeking health care in transitional countries [11]. A well-structured CHW program has already been established in Pakistan, known as the Lady Health Worker Programme (LHW-P) [12]. Lady health workers (LHWs) and lady health supervisors (LHSs) will be collectively referred to as CHWs or health workers in this protocol. The LHW-P covers 85% of the rural population in Pakistan through 115,000 LHWs. They act as “focal points of care” for their assigned catchment areas [13]. LHWs are required to have a minimum of 8 years of education and receive 15 months of training. They maintain health records and track basic health indicators [14]. An LHW typically serves between 100 and 150 houses (1000 people) per month [15] and provides health promotion and disease prevention counseling and monitoring for vaccinations, family planning, and maternal and child health care along with appropriate referrals to specialists where needed [16]. Each LHW is supervised by an LHS. LHSs have higher educational and competency levels as compared to LHWs and are tasked with coordination, monitoring, and supervision of the LHWs at the level of union council (smallest administrative unit of the district). Typically, each LHS oversees 20-25 LHWs and provides them with the required supervision and mentorship. LHSs report to their District Health Officer or District Coordinator [14].

In Pakistan, LHWs can be used to deliver mental health care by acting as the first responders to screen, counsel, and make suitable referrals. Mental health interventions offered by LHWs can provide cost-effective health promotion preventive options for communities with limited resources [17]. The stigma associated with seeking mental ill-health can be reduced if LHWs are tasked to provide mental health care at community doorsteps. Previously, engaging CHWs has been shown to improve health outcomes for heavily stigmatized disorders such as schizophrenia [18].

The role of mobile and wireless technologies has an immense capacity to promote and strengthen health care through CHWs. These innovations use the ubiquity of mobile phones to enhance the functionality of health systems [19]. Mobile penetration being greater than 90% in LMICs has strengthened the viability of mobile health (mHealth) programs, especially in remote areas [20].

Besides being cost-effective, mobile technology has better potential for extensive population-based outreach given its higher penetration and accessibility [21,22]. App-based interventions offer early detection of symptoms, decrease barriers associated with traditional in-person interventions, and offer efficient use of time by minimizing delays in initiating contact with the health care system and self-pacing. It no longer remains limited by proximity to available psychotherapists [23-25].

The promising role of mHealth in screening symptoms of depression and anxiety and halting progression toward severity has been highlighted in previous work [26]. The usefulness of mobile apps for making mental health referrals was highlighted by a quasi-experimental study from India in 2020, in which CHWs were trained to screen the community participants (CPs) through a mobile app for depression and anxiety using the Patient Health Questionnaire-9 (PHQ-9) and Generalized Anxiety Disorder-7 Scale (GAD-7) and make referrals with the help of psychiatrists. The study reported a significant reduction in the depression and anxiety scores of 900 participants and this task-sharing was found useful for increasing access to mental health care in rural areas [26].

Like other transitional countries, Pakistan has also given less consideration to mental health at the policy level [27]. Hence, instead of initiating a parallel mental health system, the most convincing strategy would be the horizontal integration of mental health services within the current primary health system [28]. This would require the integration of the primary health care workforce into the agenda of the governments, nongovernmental organizations, and global mental health stakeholders [29].

The general objective of this study is to assess the feasibility, acceptability, and usefulness of a digital app-based mental health intervention (mPareshan) delivered by LHWs at community doorsteps to screen or track symptoms of anxiety and depression, offer supportive counseling to halt disease progression, and provide appropriate referrals to the next level of care.

### Aims and Objectives

The aims and objectives of this study are to (1) assess the feasibility, acceptability, and usefulness of a digital app-based intervention (mPareshan) delivered by LHWs to adult men and women in Badin District, Sindh, Pakistan; (2) examine the effect of mPareshan intervention on-screen positive (SPs) participants’ mean anxiety and depression scores; and (3) observe the effect of a contextually adapted Mental Health Gap Action Programme (mhGAP) training on mental health literacy (knowledge) and skills of LHWs and LHSs working in Badin District.

## Methods

### Study Procedure Outline

Project mPareshan is a prospective, single-district, pre- and posttest implementation research trial, using mixed methods (qualitative and quantitative) of data collection. All phases of the trial are described in detail in the following sections. In the preintervention phase, formative qualitative assessments are conducted with relevant stakeholders (CPs, LHWs, LHSs, and policy makers) to determine the acceptability and appropriateness of delivering mental health counseling via a mobile app. Following this, in a baseline prevalence survey, residents of Badin District are screened by trained data collectors for symptoms of anxiety and depression using standardized psychometric scales of GAD-7 and PHQ-9, respectively. Individuals having symptoms of mild and moderate anxiety or depression are then identified as SPs. Before commencing the intervention, LHWs and LHSs are trained so that their mental health literacy, communication, and counseling skills improve. The training content is contextually adapted from the World Health Organization (WHO) mhGAP guide 2.0 (designed for nonspecialist settings) [30]. A pre- and posttraining assessment of health workers assesses improvement in their knowledge and skills. Next, the mPareshan app is developed based on stakeholder feedback acquired during the formative qualitative inquiry. The app segments and intervention contents are described below. In the intervention phase, SPs receive 1 home-based counseling session per month by LHWs over a period of 6 months. After the intervention phase, a quantitative end line assessment of the SPs is done to assess change in anxiety and depression scores. Postintervention qualitative assessments of key stakeholders determine barriers and facilitators in intervention uptake and rollout. A schematic diagram of the study procedures and expected outcomes is presented in Table 1. This study has been prospectively registered by the Australian New Zealand Clinical Trial Registry (ACTRN12622000989741). Written informed consent is taken from all participants at each stage of the study.

**Table 1:** Summary of mPareshan implementation research methods: phase-wise data collection procedures and expected outcomes in Badin District, Sindh, Pakistan (2022-2023).

Research questions	Phase 1: preintervention				Phase 2: intervention		Phase 3: postintervention			Expected outcome
	Qualitative data	Quantitative data		Intervention rollout^a^		Qualitative data		Quantitative data		
	FGDs^b^ and IDIs^c^	HH^d^ survey, PHQ-9^e^, and GAD-7^f^	mhGAP^g^-based training	Regular feedback received through mPareshan app		FGDs and IDIs		End line survey, PHQ-9, and GAD-7		
What is the point prevalence of anxiety and depression among a sample of rural households?		✓							Establishing prevalence of mild, moderate, and severe symptoms of anxiety and depression in Badin District.	
Is it feasible to implement an mHealth^h^-based mental health intervention through LHWs^i^ at community doorsteps?	✓					✓			Acceptability of LHW or LHS^j^ for using mPareshan app in managing anxiety and depression. Perceived barriers and facilitators in using the mPareshan app.	
Is there a change in CHW knowledge and skills in assessing, diagnosing, and managing anxiety and depression because of mPareshan mhGAP-based training?			✓						Capacity building of LHS and LHWs through mPareshan training.	
Is there any change in anxiety and depression scores of SPs^k^ because of mPareshan intervention?		✓						✓	Change in anxiety and depression scores of SPs. Operability, usefulness, and task-technology fitness of mPareshan app to the end users.	

^a^Intervention described in the last figure.

^b^FGD: focus group discussion.

^c^IDI: in-depth interview.

^d^HH: household.

^e^PHQ-9: Patient Health Questionnaire-9.

^f^GAD-7: Generalized Anxiety Disorder-7 Scale.

^g^mhGAP: Mental Health Gap Action Programme.

^h^mHealth: mobile health.

^i^LHW: lady health worker.

^j^LHS: lady health supervisor.

^k^SP: screen positive.

### Study Site

Participants for the intervention are recruited from Badin, which is a coastal district in Pakistan’s southern province of Sindh with a population of 1.8 million. There are 5 Talukas (administrative units) and 49 union councils. Badin has an average literacy rate (ability to read and write) of 24% with an approximate household (HH) size of 6 persons [31]. The district has one of the highest suicide rates in the province with a poor mental health care infrastructure [32]. Badin has a functional national LHW-P with 1100 LHWs working under the supervision of 36 LHSs.

### Phase 1: Preintervention

#### Formative Qualitative Assessments

The study framework for the qualitative assessment was guided by the reach, effectiveness, adoption, implementation, and maintenance (RE-AIM) components of the RE-AIM framework [33]. The framework provides a thorough implementation research structure and focuses on several outcomes like acceptability, appropriateness, adoption, feasibility, and so forth [34].

Qualitative assessments (focus group discussions [FGDs] and in-depth interviews [IDIs]) in the initial formative phase are conducted in separate groups with various stakeholders comprising LHSs, LHWs, CPs, and policy makers. The purpose is to assess stakeholder perceptions regarding the acceptability, appropriateness, and feasibility of executing the digital intervention. Permanently employed LHWs and LHSs who regularly perform HH visits and report to their program supervisors are randomly selected for the interviews. CPs are influential people of the community (religious scholars and teachers) capable of providing their viewpoints. Policy makers who are key decision makers at the Provincial Department of Health and the Provincial Program Implementation Unit of LHW-P, Badin are selected for IDIs. Thus, information and feedback obtained before rolling out the intervention are helpful in designing the various features of the mPareshan app (the main intervention).

A semistructured guide helps to carry out the FGDs and IDIs. The guide has a preset list of open-ended questions, organized in a logical pattern with relevant probes (Multimedia Appendix 1). The probes explore the acceptability and appropriateness of involving LHWs in this technology-driven intervention. This guide is translated into the local language, Sindhi, and then translated back into English. Qualitative interviews last about 30-45 minutes, or until the point of saturation. All participants provided written consent before starting the interview.

#### Baseline Quantitative Assessments

##### HH Survey

A baseline survey was conducted in 5 Talukas of Badin District, Sindh to identify individuals having symptoms of anxiety and depression. Trained field staff visit HH for data collection. Data collectors are provided training on administering the GAD-7, PHQ-9, and entering data electronically using REDCap (Research Electronic Data Capture; Vanderbilt University) on a tablet. Data are also collected on basic sociodemographic variables.

The GAD-7 scale is used to screen symptoms of anxiety disorders. This scale has 7 items, and each item is rated on a sliding scale of 0-3 based on the frequency of occurrence of the symptoms. The maximum possible score is 21 and the minimum score is 0. Scores of 5, 10, and 15 are taken as the cutoff points for mild, moderate, and severe anxiety, respectively. GAD-7 has 89% sensitivity and 82% specificity for detecting generalized anxiety disorder at a cutoff score of 10 [35].

The PHQ-9 screens for symptoms of depression. There are 9 items rated on a scale of 0 to 3. The maximum possible score is 27 and the minimum score is 0. Scores 5, 10, 15, and 20 represent cutoff points for mild, moderate, moderately severe, and severe depression, respectively. PHQ-9 has 88% sensitivity and 88% specificity for detecting major depression at a cutoff score of 10 [36].

Participants with mild or moderate symptoms of anxiety and depression on the GAD-7 and PHQ-9 are identified as SPs and invited to take part in the mPareshan app–based counseling sessions.

##### Assessment of CHW’s Mental Health Literacy

**Figure 1:**
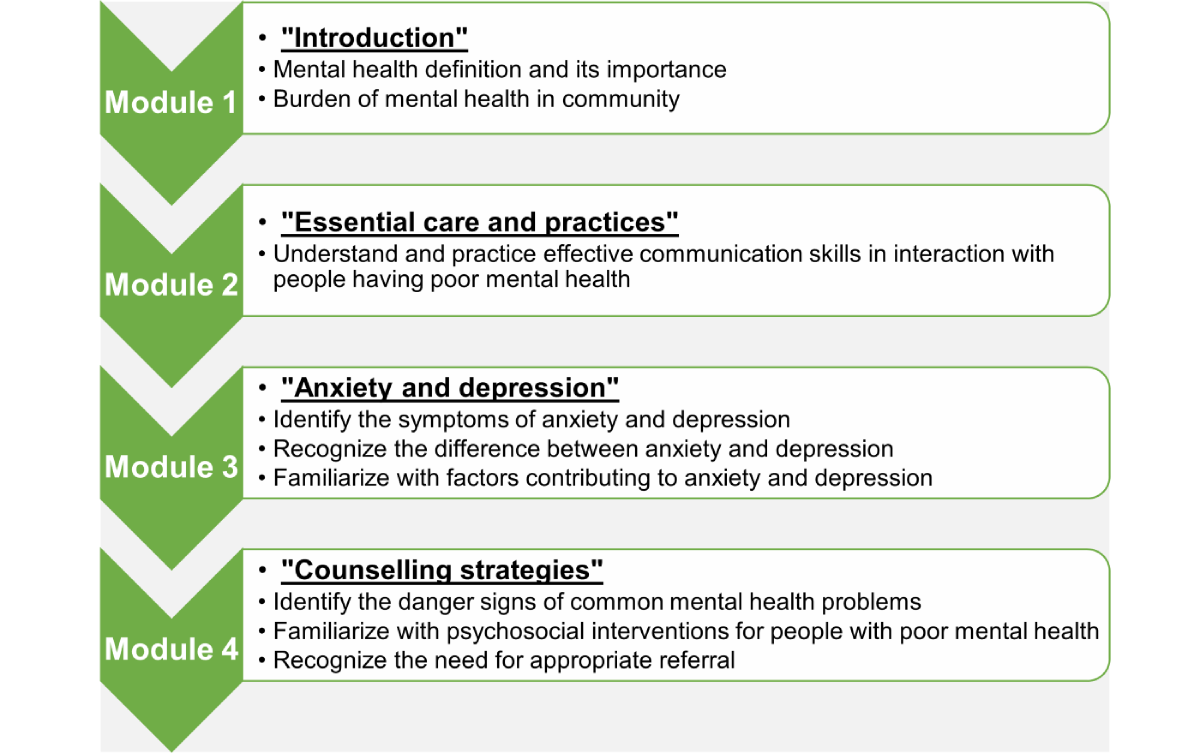
Outline of mPareshan training curriculum to improve the mental health literacy of health workers.

Before commencing the mental health intervention, a manual is designed to train LHSs and LHWs in the identification of symptoms of anxiety and depression, counseling techniques, and making appropriate referrals [37]. The brief outline of this modular training curriculum is given in Figure 1. The curriculum content is an adaptation of WHO Mental Health Gap Action Program-Intervention Guide V2.0 (mhGAP-IG 2.0) [30]. A pre- and posttraining knowledge and skills assessment of the health workers is also carried out to determine change in their mental health literacy and awareness.

### Phase 2: Intervention Rollout

#### Eligibility Criteria for Participant Enrollment in Intervention

Residents who are 18 years of age and older, who are SP for “mild” or “moderate” symptoms of anxiety or depression as determined by the GAD-7 and PHQ-9, are recruited for the mPareshan intervention. Individuals receiving therapy or pharmacological treatment for mental health issues and exhibiting severe anxiety or depression with danger signs (self-harm, harm to others, and suicidal ideation) are not included in the study.

#### Main Intervention: mPareshan Digital App

**Figure 2:**
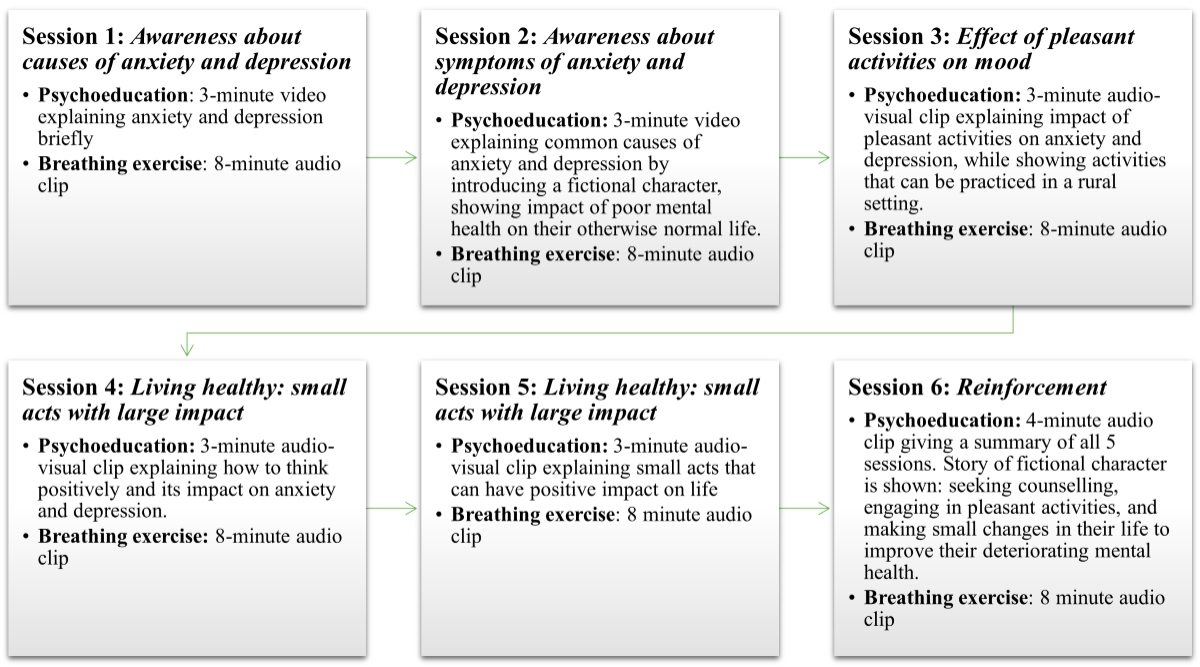
mPareshan app: the content of counseling sessions delivered by lady health workers to those who screened positive for anxiety and depression.

The mPareshan app has been designed based on feedback received from stakeholders in the formative phase. The app has 3 segments—tracking, counseling, and referral. The tracking segment records information on participant recruitment or retention and consent, and has interfaces for LHS, LHW, and study coordinator to access and record their feedback. Based on a 2-week recall, the referral segment identifies danger signs related to suicidal ideation, self-harm, and harm to others and then suggests appropriate referrals to the nearest mental health facility accordingly. In the absence of danger signs, the LHW is guided to the counseling segment of the app to deliver counseling to the SPs. This segment of the app has features of psychoeducation delivered through audio and video clips, breathing exercises, and imparts skills to cope with stress. The counseling segment has different content for each of the 6 sessions, which last around 20 minutes. An outline of the content covered in the 6 sessions is provided in Figure 2.

Prior to the commencement of the 20-minute counseling session, the LHW requests the SP to be seated in a comfortable, preferably less crowded place in the home. At the end of each session, the participant is instructed to practice breathing exercises as homework until the next session. The LHW ensures that the participant feels comfortable and consents to receiving the counseling. If the SP feels uncomfortable at any point in time, the session is discontinued.

Completion of counseling segment redirects the LHW to the section on feedback where she checks all activities that are performed in the session and records her written comments. Once submitted to the server by the LHW, the session gets locked and is passed on to her LHS for review. The LHS logs in from her portal to review all the feedback provided by the LHW and submits it to the study coordinator for a final check. The subsequent session gets unlocked for the LHW after 15 days of completion of the previous session.

These sessions coincide with LHWs’ scheduled monthly HH visits in their catchment area. The health workers assigned to the HH of SPs are trained to use configured Samsung Galaxy Tab A7 (4G; Samsung Electronics) on which the mPareshan app is downloaded. The training facilitators demonstrate how health workers are to operate tracking, referral, counseling, and feedback segments of the mobile app.

**Figure 3:**
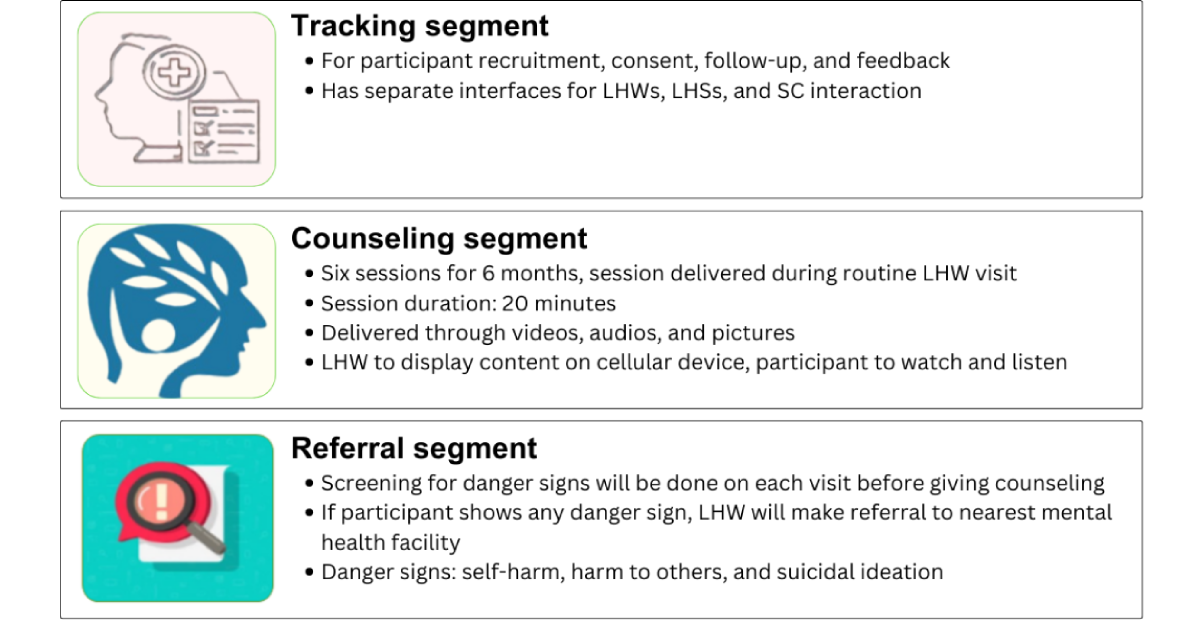
Overview of mPareshan app segments. LHS: lady health supervisor; LHW: lady health worker; SC: study coordinator.

**Figure 4:**
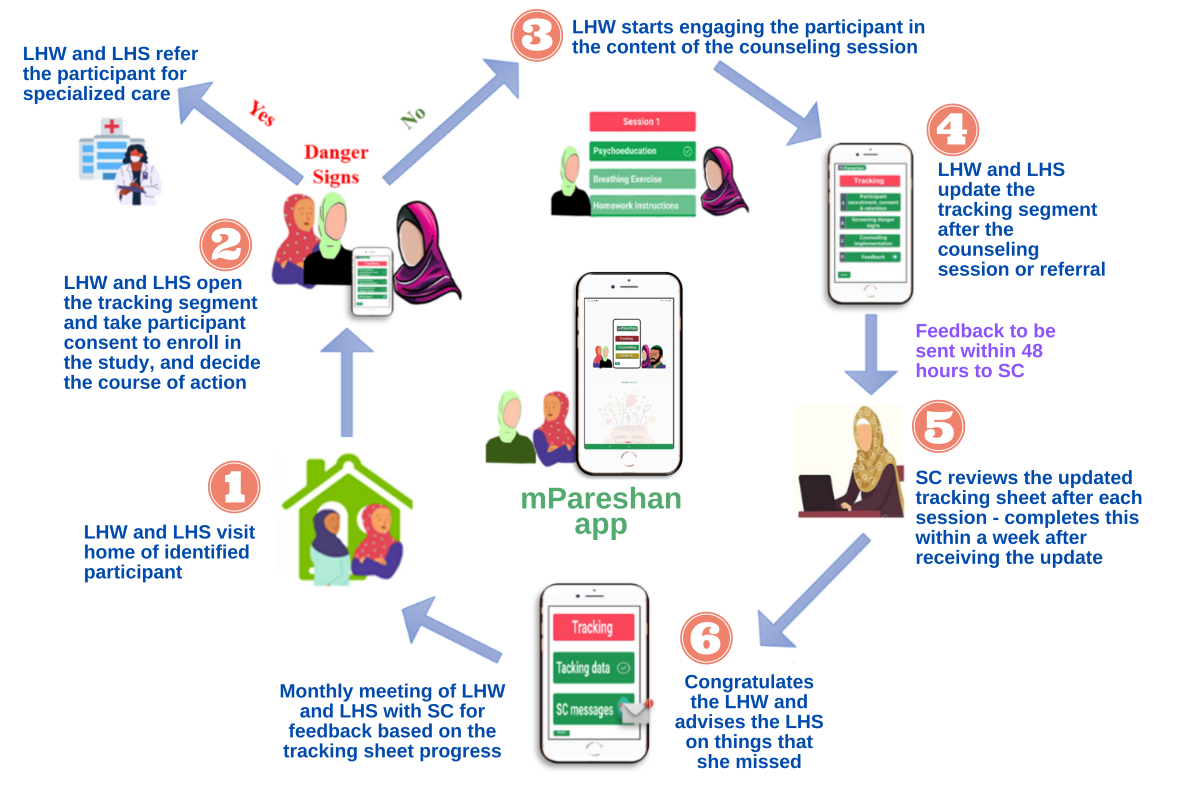
mPareshan intervention workflow steps for implementation roll out. LHS: lady health supervisor; LHW: lady health worker; SC: study coordinator.

A summary of the app layout and project workflow (implementation steps) is presented in Figures 3 and 4, respectively.

### Phase 3: Postintervention

#### End Line Quantitative Assessment

After the conclusion of the sixth mental health counseling session, an end line HH survey is conducted by trained data collectors using the same PHQ-9 and GAD-7 psychometric tools. This determines the change in mean symptomatic scores of anxiety and depression among intervention recipients (SPs), indicating intervention effectiveness (clinical outcome).

#### End Line Qualitative Assessment

In the postintervention phase, qualitative assessments (FGDs and IDIs) are done to ascertain the feasibility of intervention uptake and understand facilitators and barriers in implementation rollout. Key stakeholders for FGDs include those randomly selected LHSs and LHWs who took part in the delivery of the intervention. Moreover, for IDIs, CPs in the postintervention qualitative phase also include those individuals who receive the digital counseling intervention, namely, the SPs. All participants provided written informed consent prior to their interviews.

### Sample Size Calculations

For the baseline screening survey, the sample size is calculated using OpenEpi (version 3.01; Emory University) [38]. Assuming a 30% prevalence of depression and anxiety symptoms among adults, at a 5% level of significance and 80% power, 323 people are required to assess point prevalence of depression and anxiety [39]. Catering to 10% refusals, the final sample size is 366 individuals.

For the change in mental health literacy of CHWs, a total of 72 LHSs and LHWs are required to detect a mean difference of 1.5 (SD 3.9) in pre- and posttraining knowledge and skills scores, assuming 5% significance level and 80% study power [40].

For receiving mPareshan app-based intervention, the sample size for recruitment of SPs is calculated using Medcalculator (version 19.8; MedCalc Software). Assuming a mean difference between pre- and postintervention of PHQ-9 scores of 1.5 (SD 5.6; mean score at baseline=11.61 and postintervention=10.1), the minimum sample size required at a 5% level of significance and 80% power is 112. Considering a 10% attrition rate, with some oversampling, the expected sample size is 123 for recruiting participants for the mPareshan intervention and demonstrating a reduction in symptoms [41].

### Data Analysis

Baseline and end line data collected from respondents is being exported from REDCap to SPSS (version 21; IBM Corp). Frequency and proportion will be used to report categorical variables. Depending on the distribution of anxiety or depression scores of SPs and knowledge or skills scores of health workers, paired *t* tests or McNemar’s chi-square tests will be used to evaluate the change in outcomes. The mean difference with 95% CIs will be reported. A *P* value of <.05 will be considered statistically significant.

All interviews will be transcribed in English. Using NVivo (Lumivero), content analysis will be conducted on all IDI and FGD transcripts, and codes will be organized into either emergent or predetermined categories by the researchers. Commonalities and differences across the data will be identified and clustered around thematic sections. Verbatim quotes will be added to complement the themes. RE-AIM framework will be used to describe the feasibility outcomes [33].

### Ethical Considerations

This study protocol has been approved by the Ethical Review Committee of Aga Khan University (ERC# 2021-6570-20015). mPareshan is an implementation research feasibility trial and is not a clinical or drug trial. No adverse events are, therefore, anticipated. Any participants exhibiting (the a priori–defined) danger signs will be immediately referred during participant recruitment or counseling phases. Written, informed consent is obtained from each participant in their local language at each phase of the study. Data are anonymized and participant confidentiality is maintained. No compensation is offered to SPs for participation in the counseling sessions.

## Results

Data were collected in 2022-2023. At the time of this submission (April 2024), we are analyzing the results of 366 individuals who participated in the baseline prevalence survey, the change in knowledge and skills of 72 health workers who took the mPareshan training, change in anxiety and depression scores of 98 SPs recruited for app-based counseling intervention, and stakeholder perceptions gathered from 8 FGDs and 18 IDIs as part of qualitative assessments. Final data cleaning and data analysis are ongoing and the results will be disseminated through peer-reviewed publications in 2024.

## Discussion

Project mPareshan will assess the feasibility of using frontline workers in early home-based screening of anxiety and depression, providing app-based mental health counseling and prompt referrals. The community will ultimately benefit as the mental well-being of the population improves by a projected decrease in mean symptomatic scores of anxiety and depression. The contextually adapted mhGAP training for health workers will also lead to an improvement in their mental health literacy thus building the capacity of the primary health care workforce in mental health service delivery.

Previous studies using mHealth modalities have used cellular devices for the purpose of making referrals and record-keeping [26]. Despite the high reported suicide rate, there is a lack of awareness about mental health and the availability of specialized mental health services is suboptimal in Badin District. The novel approach being tested in this trial is expected to improve the mental health of rural populations where access to health care is limited and difficult. Results from this study will be important in initiating a policy dialogue for horizontal integration of basic mental health counseling initiatives in the current primary health system of low-resource settings.

Implementation challenges in this trial in the field included partial disruption and delay in intervention execution attributed to natural disasters, for example, floods in 2022. Furthermore, fluctuations in internet connectivity partially compromised the delivery of the app-based counseling sessions initially. The current substantial workload of LHWs makes it difficult to add mental health counseling as part of their routine package of service delivery. Moreover, considering the small sample size of participants enrolled for receiving the intervention, the generalizability of study findings may be an issue.

Based on the anticipated results of this pilot project, mental health can be introduced as part of the LHW curriculum and service delivery package. This will set the stage for integrating mental health into primary health care.

## Appendix

Multimedia Appendix 1 Guide for focus group discussions and in-depth interviews used for qualitative assessments to assess feasibility and uptake of intervention.

## Abbreviations

CHW: community health worker
CP: community participant
FGD: focus group discussion
GAD-7: Generalized Anxiety Disorder-7 Scale
HH: household
IDI: in-depth interview
LHS: lady health supervisor
LHW: lady health worker
LHW-P: Lady Health Worker Programme
LMIC: low- and-middle income country
mHealth: mobile health
mhGAP: Mental Health Gap Action Programme
mhGAP-IG 2.0: Mental Health Gap Action Program-Intervention Guide V2.0
PHQ-9: Patient Health Questionnaire-9
REDCap: Research Electronic Data Capture
RE-AIM: reach, effectiveness, adoption, implementation, and maintenance
SP: screen positive
WHO: World Health Organization

## Data Availability

The data sets generated and analyzed during this study will be available from the corresponding author on reasonable request. It is to be noted that additional data pertaining to measurement of loneliness was also collected on the University of California, Los Angeles (UCLA) Loneliness Scale (LS-10) at the time of baseline and end-line household survey and will be reported separately.
