# Supplementary material for: Technology-Assisted Mental Health Intervention Delivered by Frontline Workers at Community Doorsteps for Reducing Anxiety and Depression in Rural Pakistan: Protocol for the mPareshan Mixed Methods Implementation Trial
Source: JMIR Res Protoc. 2024 Jul 23;13:e54272. doi: 10.2196/54272 (PMC11303881; doi:10.2196/54272)
Supplement: Multimedia Appendix 1 [file resprot_v13i1e54272_app1.docx]

| Sections | Themes | Main probes |
| --- | --- | --- |
|  |  |  |
| 1 | Burden and determinants of mental health | - Perceptions of participants about mental health burden and its determinants. - Availability of mental health services in the community. |
| 2 | Acceptability and appropriateness of delivering a mental health intervention | - Rapport of LHWs as front-line health care providers in rural areas - LHW-P capacity to address mental health. - Capacity of LHWs to deliver mental health intervention - Acceptability of intervention among policy makers, LHWs and community |
| 3 | Adoption and task-technology shift of mHealth intervention delivered by LHWs | - LHSs awareness regarding android phone technology for provision of mHealth services. - Willingness of LHWs to provide mental health services utilizing technology-assisted app. |
| 4 | Uptake of intervention | - Experience of Health Workers and community participants involved in intervention - Views regarding app features, usage, and content - Views on the benefits of intervention. |
| 5 | Barriers to implementation roll-out and sustainability | - Barriers experienced by stakeholders during intervention roll out. - Suggestions for improvement and sustainability. |
| 6 | Facilitators in implementation roll-out | - Factors that facilitated implementation roll-out - Importance of LHSs as supervisors of LHWs |

LHW: Lady Health Workers, LHW-P: Lady Health Worker Programme, LHS: Lady Health Supervisor
